# Research Progress on Metal–Organic Frameworks by Advanced Transmission Electron Microscopy

**DOI:** 10.3390/nano13111742

**Published:** 2023-05-26

**Authors:** Anqi Zheng, Kuibo Yin, Rui Pan, Mingyun Zhu, Yuwei Xiong, Litao Sun

**Affiliations:** SEU-FEI Nano-Pico Center, Key Laboratory of MEMS of Ministry of Education, Southeast University, Nanjing 210096, China

**Keywords:** metal–organic frameworks, transmission electron microscopy, in situ TEM, structural characterization, structure–activity, dynamics visualization

## Abstract

Metal–organic frameworks (MOFs), composed of metal nodes and inorganic linkers, are promising for a wide range of applications due to their unique periodic frameworks. Understanding structure–activity relationships can facilitate the development of new MOFs. Transmission electron microscopy (TEM) is a powerful technique to characterize the microstructures of MOFs at the atomic scale. In addition, it is possible to directly visualize the microstructural evolution of MOFs in real time under working conditions via in situ TEM setups. Although MOFs are sensitive to high-energy electron beams, much progress has been made due to the development of advanced TEM. In this review, we first introduce the main damage mechanisms for MOFs under electron-beam irradiation and two strategies to minimize these damages: low-dose TEM and cryo-TEM. Then we discuss three typical techniques to analyze the microstructure of MOFs, including three-dimensional electron diffraction, imaging using direct-detection electron-counting cameras, and iDPC-STEM. Groundbreaking milestones and research advances of MOFs structures obtained with these techniques are highlighted. In situ TEM studies are reviewed to provide insights into the dynamics of MOFs induced by various stimuli. Additionally, perspectives are analyzed for promising TEM techniques in the research of MOFs’ structures.

## 1. Introduction

Metal–organic frameworks (MOFs) are porous crystalline materials composed of inorganic metal nodes and organic ligands [1]. As a unique class of materials featuring tunable topologies, large specific surface areas, and adjustable chemical compositions, MOFs are promising candidates for catalysis [2], gas storage and separation [3,4], energy storage and conversion [5], chemical sensing [6], water adsorption [7], and lithium-ion storage [8]. Structure–activity relationships can guide the rational design and applications of flexible and functional MOFs. Atomic-scale determination of the crystal structures is a well-founded prerequisite for understanding the relationships. Surfaces, interfaces, defects, and host–guest interactions are the main microstructures, which directly affect the properties of MOFs. Surfaces influence the surface-related properties and growth processes [9,10]. Interfaces are of great significance for plasma crystals, composites, MOF-based devices, among other applications [11,12,13]. Defects provide a method to tune the porosity locally and to create active open metal sites for MOFs [14,15], which allows for defect engineering of the specific function [16,17,18]. Guest species, including ions, particles, clusters, and molecules, accommodate in ideal frameworks of MOFs crystals with distinctive porosity and long-range orderly structures [19,20,21]. In addition to the intrinsic properties of the static structure, a thorough understanding of the growth and transformation mechanisms, as well as of the evolution pathways of MOFs under working conditions is crucial to improve the applications of MOFs.

Transmission electron microscopy (TEM) is an undoubtedly unique and powerful technique to characterize atomic structures and dynamics of nanomaterials. Apart from crystal structural analysis by electron diffraction, both TEM mode and scanning transmission electron microscopy (STEM) mode permit direct imaging of regions of interest in the specimen, including its periodic, non-periodic, local, and porous details inside and on the surface of MOFs at the atomic scale [22,23,24]. Meanwhile, TEM is capable of being combined with spectroscopic techniques to examine the chemical elements [25]. Moreover, by using novel in situ sample holders, TEM can introduce external fields and conditions such as low temperatures, heating, biasing, liquids, and gases in real-time, which allows for on-demand scenarios of the sample in practical applications [26].

However, using TEM for characterization or as a research platform for MOFs is challenging due to the extreme sensitivity of MOFs to electron-beam irradiation. This fundamental hinderance of MOFs originates from their organic components and the coordination bonds that link organic parts to the metals. It results in either structural decomposition before the detection completion or in a lack of intrinsic characteristics during acquisition [22,27]. Therefore, the feasibility and utility of research using TEM is limited due to the nature of MOFs. Interestingly, several TEM-based methods and techniques have been developed to make them suitable for beam-sensitive materials. The idea of low electron dose and low temperature was established under the consideration of damage mechanisms in MOFs [28,29]. A variety of advanced TEM techniques have been developed, mainly including three-dimensional electron diffraction (3DED), imaging using direct-detection electron-counting (DDEC) cameras, and integrated differential phase-contrast scanning transmission electron microscopy (iDPC-STEM) [30,31,32].

Several review articles have discussed work on the study of MOFs using TEM-based techniques. Huang et al. summarized the development of 3DED methods and demonstrated their capabilities for structural characterization of MOFs [33,34]. Wiktor et al. [35] and Liu et al. [36] presented the problems and approaches in the research of MOFs by TEM, and the progress made in the structural characterization of MOFs until then. Gong et al. [37] and Zhang et al. [38] subsequently discussed new findings based on in situ TEM methods. However, no articles have comprehensively proposed the scope of applicability of various TEM-based techniques for the investigation of MOFs materials from the perspective of theoretical principles and practical operations. Herein, we discuss the challenges, strategies, and advanced techniques for MOFs research based on TEM, and introduce some representative and new research advances in structural analysis and dynamic evolution. We demonstrate the practicability and indispensability of TEM as a powerful tool in this field. Meanwhile, we propose some perspectives on cutting-edge TEM-based techniques that have potential for MOFs study.

## 2. Challenges and Chances of TEM Studies

Structural characterization of MOFs via TEM is challenging because MOFs are so unstable and sensitive to the high-energy electron beam. This requires an understanding of electron-beam damage mechanisms in MOFs.

### 2.1. Damage Mechanisms

The radiation damage mechanisms of MOFs under the high-energy incident electron-beam mainly include radiolysis, knock-on damage, and thermal effects [22]. In practical situations, identifying the predominant mechanism can help determine the proper method to minimize damage [27].

Radiolysis (or ionization damage) is the ionization of specimen atoms by electron–electron interactions via inelastic scattering, resulting in chemical bond weakening or breakage. It is the main cause of the reported degradation of MOFs, especially at lower voltages. High-energy incident electrons may mitigate this beam damage by decreasing inelastic scattering events. However, the signal-to-noise ratio of the image and spectrum is not improved because both the inelastic and the elastic cross-sections are inversely proportional to the incident energy [39]. Low temperature effectively improves the beam stability of the specimen and reduces radiolysis. This can be achieved by cooling the sample areas in TEM to cryogenic temperatures using liquid nitrogen or helium (cryo-TEM) [27,29].

Knock-on damage results from direct electron–nucleus interactions, specifically atomic displacements or sputtering in the original specimen caused by high-energy electrons. Low-energy incident electrons prevent knock-on damage, but this comes at the cost of weak beam penetration depth and poor signal resolution [22]. This problem can be alleviated by the reduction of the TEM accelerating voltage below the sample-specific threshold value without loss of resolution (low-voltage TEM).

Thermal effects (or beam heating) result from collective crystal lattice vibration caused by electron–atom interactions, and can be mitigated by lowering the incident-beam current [39]. Cryogenic temperatures can partially alleviate this damage.

In addition to structural disintegration [40], materials with poor electrical conductivity are charged by electron beams, causing image blurring due to image drift and vibration [41].

### 2.2. Strategies for Minimizing Damages

Low-dose TEM and cryo-TEM could be used for probing the microstructure of MOFs by TEM without damaging their intrinsic properties.

#### 2.2.1. Low-Dose TEM

Reducing the electron dose (low-dose TEM) is a general solution applied to MOFs regardless of the damage mechanism, given that all these electron beam-induced irradiation damages in MOFs are dose dependent. A preliminary assessment is required to determine whether the low-dose conditions are within the acceptable range to maintain the crystallinity stability. Electron diffraction (ED) is an effective and feasible way to determine the electron dose that the MOF can withstand. The ED pattern varies with the increasing of electron dose, indicating the changes in the structure and the appearance of disordered phases. Shorter exposure time and lower intensity can achieve low electron doses [28]. The maximum electron dose that MOFs can withstand depends on the materials and the TEM operating conditions. Taking typical MOFs in TEM mode under 300 kV accelerated electron beam as examples, the electron dose that MIL-101(Cr) can withstand is ~16 e^−^ Å^−2^ [42]. The onset of beam damage for UiO-66(Zr) was from 10 to 20 e^−^ Å^−2^ [22], and ZIF-8(Zn) was about 25 e^−^ Å^−2^ [43]. Compared to the static characterization, in situ TEM observations require a longer irradiation time, which necessitates an electron dose well below the damage threshold to ensure the integrity of the sample region of interest [44].

#### 2.2.2. Cryo-TEM

Cryo-TEM contributes to probe structural details of MOFs by preserving their stability over prolonged irradiation times [22,29]. This is because cryogenic temperatures diminish radiolysis and compensate for thermal effects to a certain extent. In the absence of advanced imaging and signal acquisition methods, high-resolution TEM (HRTEM) at liquid nitrogen temperature imaged the complete pore structure and the crystal lattice periodicity of MOF-5(Zn) nanocrystals [29]. Cryogenic temperatures also allowed the elucidation of the ordered internal architecture of large-area conductive 2D Cu_2_(TCPP) (TCPP = meso-tetra(4-carboxyphenyl)porphine) MOF films on dielectric substrates by HRTEM and ED [45]. Furthermore, ED determined the structures of the highly porous CAU-7(Bi) at 120 K [46].

**Figure 1:**
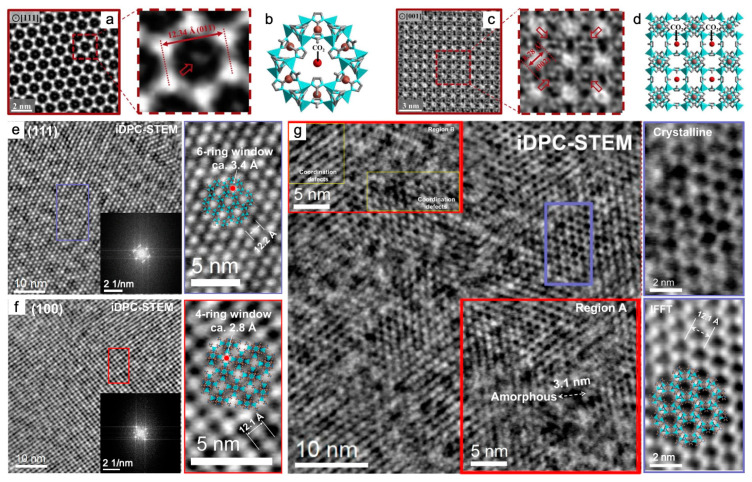
HRTEM characterization under cryogenic temperatures of CO_2_@ZIF-8(Zn) [49] and protein-ZIF-8(Zn) composites [51]: (**a**–**d**) Host–guest structures within ZIF-8(Zn) viewed along <111> (**a**,**b**) and <001> (**c**,**d**) projection. (**a**,**c**) Left: CTF-corrected Cryo-TEM images of CO_2_-filled ZIF-8 particle. Middle: magnified image of a single ZIF-8-unit cell. HRTEM images with red dashed boxes in the middle are magnified images of the red dashed regions from images with red solid boxes on the left. Red arrows indicate density near the center of the unit cell. (**b**,**d**) Simulated structure of ZIF-8 with DFT-predicted binding site of CO_2_ indicated by red spheres in (**a**,**c**), respectively. Reprinted with permission from [49]. Copyright 2019, Elsevier; and (**e**–**g**) iDPC-STEM images, corresponding FFT, and the structural analysis of the selected region (indicated by the same color) of BZIF-8-S from the <111> (**e**) and <100> (**f**), and BZIF-8-B from the <111> (**g**) zone axis. Red boxes and yellow boxes within the red boxes in (**g**) highlight the coordination defects and amorphous regions, respectively. Reprinted with permission from [51]. Copyright 2022, Springer Nature.

Apart from minimizing structural damage and improving electron tolerance during characterization, cryo-TEM is capable of freezing specimens in the state of interest during an in situ TEM observation. Examples include in situ studies and corresponding ex situ examinations of the crystallinity of MOFs in the liquid phase at specific reaction stages [47,48] and the construction of MOFs after interaction with gas species [49]. Ex situ HRTEM images revealed two preferred adsorption sites for CO_2_ in ZIF-8(Zn) at ~103 K, with a cumulative electron flux of ~7 e^−^ Å^−2^ using a DDEC camera (Figure 1a–d). Bright regions correspond to mass density. In CTF-corrected images with an electron dose rate of ~4.5 e^−^ Å^−2^ s^−1^ for 1.5 s, contrast near the center of the 6-ring window (Figure 1a) and 4-ring window (Figure 1c) is observed, respectively, for multiple unit cells. The density at the center indicated by red arrows likely corresponds to CO_2_ adsorbed within ZIF-8. In Figure 1c, only the pore cavity at the vertices of the 4-ring window contains this density. The unit cell of ZIF-8(Zn) expanded by ~3% due to molecular guests [49].

In addition, cryogenic conditions are well suited for the biological field, including biomacromolecule-metal–organic frameworks (biomacromolecule-MOFs). The amorphous precursor phase in the nucleation of protein-ZIF-8(Zn) was directly observed. This revealed a non-classical nucleation approach of dissolution-recrystallization and protein-rich amorphous solid phase transformation [50]. Furthermore, cryogenic temperatures allowed for an understanding of their nanoarchitectures at the atomic level. The structural difference resulted from different crystallization pathways in synthetic scenarios and significantly affected the bioactivity (Figure 1e–g). BZIF-8-S heterogeneously crystallized by solid-state transformation of the biomacromolecule amorphous phase at the spontaneously growing ZIF-8 crystal surface. High crystallinity with ~3.4 Å 6-ring window and ~2.8 Å 4-ring window in Figure 1e,f shows the crystal structure with regularly narrow pore windows that hindered the diffusion of the catalytic substrate. BZIF-8-B crystallized around the surface directly via electrostatic interaction with the induction of biomacromolecules. The crystalline, amorphous phase, and coordination defects in Figure 1g slowed the crystallization rate. These defects effectively enhanced the bioactivity [51].

## 3. Strategies, Techniques, and Research Advances

### 3.1. Traditional and Advanced Electron Diffraction

Occasionally, large MOF crystals are difficult to prepare, and only nano- or submicron-sized crystals can be produced due to the reaction kinetics and thermodynamics. Because of its ability to probe nanosized crystals, electron diffraction (ED) is better suited for structural studies of MOFs than conventional X-ray diffraction (XRD) which is ideal for atomic-scale analysis of large crystals (at least microns in size) [52]. ED is to some extent preferable to HRTEM because it requires a lower electron dose to achieve the same level of resolution. The advantage exists provided that the resolution and region of interest are within practical requirements. This supports the use of ED for the determination of the maximum amount of electron flux that MOFs can tolerate. For instance, ED reviewed ZIF-8(Zn) crystals with enough contrast at a dose rate of only ~1 e^−^ Å^−2^ s^−1^ [43]. To obtain accurate structural information, ED requires the crystal to be tilted along specific zone axes. However, beam-sensitive MOFs suffer from the time-consuming procedure to collect a few ED patterns along the precisely aligned crystallographic zone axes. The method of merging multiple ED maps requires the correct handling of multiple scattering effects. This poses a challenge to the accurate description of the crystal structure by ED [53]. Therefore, the limitations primarily result from the data collection strategy.

**Figure 2:**
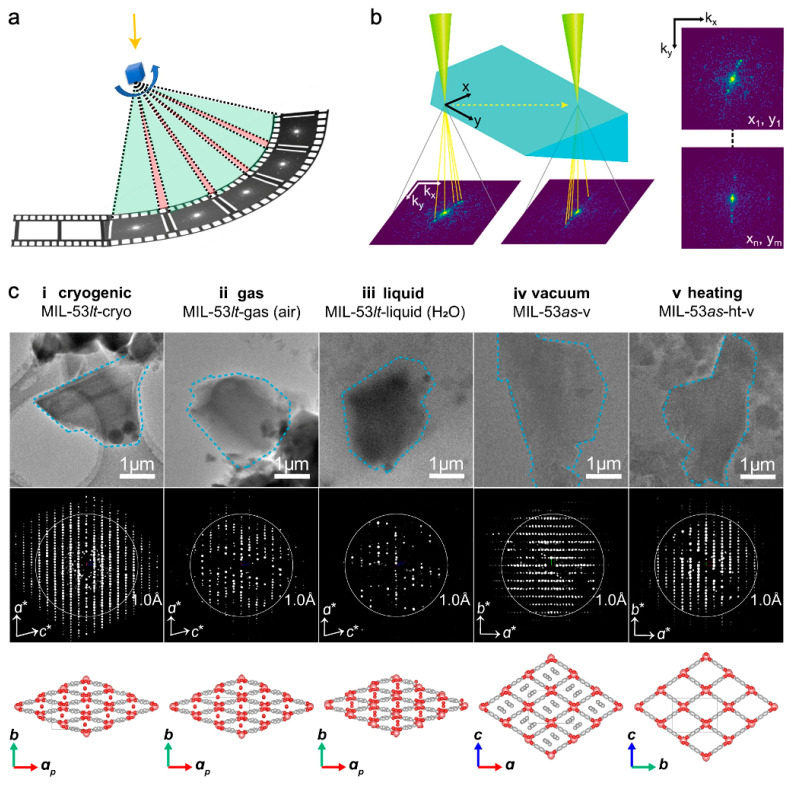
Advanced electron diffraction: three-dimensional electron diffraction (3DED) [34,69] and scanning electron diffraction (SED) [15]: (**a**) The data collection procedure of 3DED. Green: obtained data; pink: missing wedges; blue cube: target crystal; blue arrow: rotation direction; yellow arrow: incident electron beam. Reprinted with permission from [34]. Copyright 2023, Springer Nature; (**b**) Illustration of SED applied to a defect-engineered UiO-66(Hf) particle. A 2D (*k_x_*, *k_y_*) diffraction pattern is recorded in transmission at each probe position in a 2D (*x*, *y*) scan. Reprinted with permission from [15]. Copyright 2020, American Chemical Society; and (**c**) ETEM images (**top**), ETEM 3DED data (**middle**), and determined structures (**bottom**) of MIL-53(Al) single crystals under different conditions. Particles marked with blue dashed lines (**top**) were used to collect 3DED data. White circles in diffraction data (**middle**) represent a resolution of 1 Å. The 3DED data are projected along the <010> (**i**–**iii**) and <001> (**iv**,**v**) directions of the samples. (**i**) MIL-53*lt* in the low temperature phase prepared by plunge-freezing and cryogenic transfer protocols. (**ii**) MIL-53*lt* in static air (0.1 MPa). (**iii**) MIL-53*lt* covered with liquid water. (**iv**) The as-synthesized phase MIL-53*as* under high vacuum state. (**v**) The phase after calcination of MIL-53*as* at 603 K and high vacuum for 2 h. a*, b*, c* and *a*, *b*, *c* denote the axes in reciprocal space and in real space, respectively. *a_p_* indicates the projected direction of the *a*-axis. Reprinted with permission from [69]. Copyright 2022, Springer Nature.

Three-dimensional electron diffraction (3DED) allows for effective ED data collection as well as subsequent ab initio structure determination and analysis [30]. The 3DED dataset of a single crystal is a sequence of ED patterns successively recorded at different tilt angles of the TEM goniometer. The corresponding diffraction peaks were then produced (Figure 2a) [34]. This technique has evolved from stepwise to the faster continuous strategies in terms of data collection.

Among the former types, electron diffraction tomography (EDT) was first proposed in the late 2000s [54]. The program-controlled TEM sample stage automatically records the stepwise tilt angle relative to the electron beam and collects the rotation of the ED patterns. Apart from MOFs, the EDT technique has also been applied to guest distributions in MOFs, such as TiO_2_ in MIL-101(Cr) mesopores [23]. Automatic diffraction tomography (ADT) was later developed to decrease the required electron-beam intensity. ADT is used in combination with precession electron diffraction (PED) equipment to control the tilt angles of goniometers. PED is suitable for near-kinematical data collection from single zone axes. This protocol facilitates the analysis of porous and organic sub-microcrystalline samples at the single crystal scale but operates entirely in STEM mode [54,55,56]. Using ADT assisted by a cryo-TEM holder, it reconstructed a bismuth-based MOF Bi(BTB) (BTB = 1,3,5-benzenetrisbenzoate) (also denoted as CAU-7). A twinning law was proposed for the pseudohexagonal symmetry of the rodlike aggregates [46]. Compared to PED method, rotational electron diffraction (RED) contributes to the complete collection of 3DED data and reconstructs the reciprocal space with a high resolution [57]. In addition to the ADT-controlled goniometer tilt, RED also combines fine electron-beam tilt. Combined control of the two tilts further accelerates data collection and eliminates the need for precise alignment to specific zone axes. RED assisted in the direct determination of the pore structure of UiO-66(Zr), which was confirmed by Rietveld refinement of XRD [52].

The latter type of strategy allows continuous crystal rotation to speed up data acquisition while still obtaining relatively accurate and complete ED intensities as a movie. This type of strategy improves on the previous one by limiting the goniometer tilt. It is particularly suitable for crystals with low symmetry. Continuous rotation electron diffraction (cRED) has resolved the structures of several types of MOFs. It has also always supplemented relatively ambiguous XRD data in reported work. Examples include atomic positions of Ti_8_Zr_2_O_12_(COO)_16_ cluster-based PCN-415 and PCN-416 [58], and lattice of Ti^VI^ 4,4′-biphenyldicarboxylate (bpdc^2−^) MOF termed COK-47 [59], Zr chain-based PCN-226 [60], ZIF-EC1(Zn, Co) [61]. UU-100(Co) was solved to have a tetrahedral unit cell with the lattice parameters a = b = 27.3 Å and c = 19.6 Å, a possible *P*4*/mbm* space group, and rectangular channels with elliptical pores [62]. Due to their porous structures, MOFs with their enhanced conductivity have been welcomed in fields such as electrocatalysis and charge storage [63]. Electrically conductive 2D MOFs have attracted attention for their hexagonal 2D lattices, such as 2D van der Waals stacked materials. In regard to a class of 2D π-conjugated MOFs, the structural details of M_3_HHTT_2_ (HHTT = 2,3,7,8,12,13-hexahydroxy tetraazanaphthotetraphene, M = Cu^2+^ or Ni^2+^) with a resolution of ~1.5 Å convincingly verified the π-stacking by an interlayer distance of 3.19 ± 0.02 Å, resulting in a rare eclipsed AA stacking [64]. The crystal tracking technique was developed to defocus every few ED patterns in order to address the potential deviation of the crystallites from the selected regions during the continuous rotation [65]. Micro-electron diffraction (MicroED) is a technique using an ultra-low electron dose at cryogenic temperatures and is similar in principle to PED [66,67]. The applicable range has been extended from biomacromolecules to beam-sensitive materials. MicroED data from a single crystal of ZIF-8(Zn) was refined to 0.87 Å [68]. Notably, the environmental 3DED method, couples continuous 3DED and environmental transmission electron microscopy (ETEM) has been proposed and validated to study the structural dynamics under external stimuli of single microcrystals. The atomic-level ab initio structure determination was performed on MIL-53(Al) samples in various states, including the as-made phase (MIL-53*as*) and the H_2_O-containing phase (MIL-53*lt*, room temperature, in the air) [69]. Thus, it was demonstrated that in situ ED, as an in situ TEM-based approach, can also be used for analysis of the dynamic behaviors of MOFs in external fields.

Conventionally, ED is only applicable to reveal the average spatial information, such as periodic crystal structures or abnormal macroscopic features. It is exemplified by the elucidation of **reo** topology with the existence of missing cluster defects of UiO-66 (Hf) via multiple diffraction behaviors, including ED [17]. Organic molecules in pores were for the first time localized by cRED with a resolution of 0.83–1.00 Å, in the study at 96 K of [Co_2_(Ni-H_4_TPPP)]·2DABCO·6H_2_O (DABCO = 1,4-diazabicyclo[2.2.2]octane) designated by Co-CAU-36 [70]. Hydrogen atoms in ICR-1, ICR-2, and ICR-3 based on the ligand PBPA (phenylene-1,4-bis(methylphosphinic acid)) were positioned by the full dynamic diffraction theory in the least-squares refinement of the EDT [71]. Moreover, the emerging technique of scanning electron diffraction (SED) based on four-dimensional scanning transmission electron microscopy (4D-STEM) was proposed to overcome the limitations of ED (Figure 2b) [15]. Two-dimensional diffraction patterns scanned on every part of the entire sample directly reflected the size, morphology, local orientation, and spatial distribution of defect nanodomains **reo** of single-crystal UiO-66(Hf) octahedral particles. The spatial resolution of 2–5 nm was realized by a focused electron probe with a convergence angle of about 1mrad. The blocky lamellar morphology caused by the local fluctuation of phthalate concentration preferentially extended in a direction perpendicular to the {111} crystal plane. There were interfaces between the **reo** and **fcu** domains on the {211} plane. Despite the progress made, the inherent deficiency of ED in probing local structures makes TEM and STEM imaging still indispensable tools for resolving the structures of MOFs.

### 3.2. TEM and DDEC Camera

The ability of TEM imaging to directly visualize atomic-scale information has been greatly enhanced by advances in spherical aberration (Cs) correctors and contrast transfer function (CTF) correction techniques [14,72]. In terms of minimizing the electron dose during imaging, an efficient way to improve camera data acquisition is to use highly sensitive scintillators and optics fibers. These promote a high signal-to-noise ratio in the detection of signal electrons. This approach is feasible for observing MOFs [50,73]. However, in conventional cameras, the photons which are generated by the interaction of the electrons and scintillators and received by the optical fibers should be converted into an electrical signal. The conversion process consumes a significant portion of the electrons that are essential for imaging. While these cameras have excellent imaging capabilities as techniques advance, they lack the sensitivity which is needed to collect high-quality data at a very low electron-beam dose.

The DDEC camera is able to directly detect electronic signals without the signal conversion described above. This capability distinguishes the DDEC camera with an ultra-high quantum detection efficiency, which favors the detection of low-dose electrons [31]. Therefore, the DDEC camera contributes to the direct formation of the phase-contrast image with good speed and sensitivity. This is particularly suitable for low-dose and real-space imaging of hybrid materials [74]. With its ultrahigh readout speed, the DDEC camera can record nanoscale phenomena in real time at the micro- to millisecond, opening up the potential for in situ studies and accurate structural analysis. However, in order to prevent damage to internal devices, the DDEC camera has a strict electron dose limitation.

**Figure 3:**
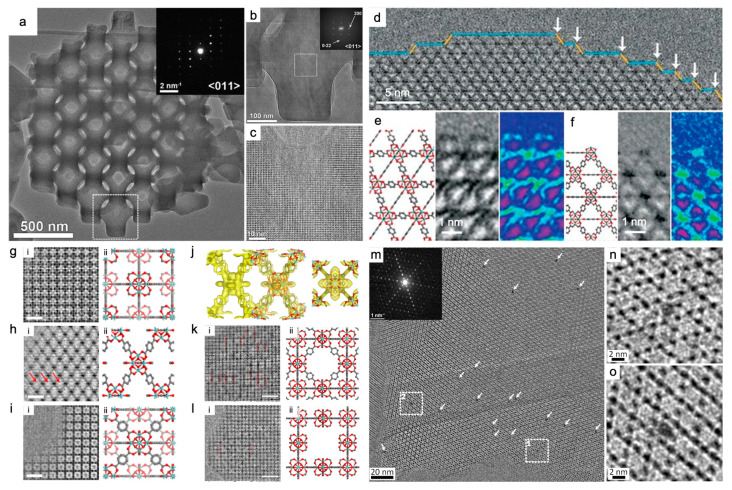
HRTEM imaging of ZIF-8(Zn) [75], UiO-66(Zr) [14,22] and Mn_12_Ac@NU-1000(Zr) [77] using DDEC cameras: (**a**–**c**) Bulk of ZIF-8(Zn) with single-crystalline nature at different magnifications taken along the <011> zone axis. The inset of (**a**) shows the corresponding ED patterns, and the inset of (**b**) shows the indexed FFT patterns; (**b**,**c**) are magnified views of the white square areas in (**a**) and (**b**), respectively. Reprinted with permission from [75]. Copyright 2018, The American Association for the Advancement of Science; (**d**–**f**) A truncation surface in a thermally treated UiO-66(Zr). (**d**) Crystal growth steps involving small {100} (labeled in blue) facets and {111} facets (labeled in yellow); (**d**) The white arrows indicate “kink” positions between {100} facets (blue) and {111} facets (yellow). (**e**) Ligand-terminated {111} surface. (**f**) Metal-terminated (ligand-free) {100}/{111} kink: (**left**) structural model; (**middle**) HRTEM image by real-space averaging; (**right**) the averaged image in rainbow colors. Reprinted with permission from [22]. Copyright 2018, The American Association for the Advancement of Science; (**g**–**l**) Missing linker defects and missing cluster defects in UiO-66(Zr); (**g**–**j**) Missing linker defects. CTF-corrected HRTEM images in column (**i**) and the projected structural model (Zr, cyan; O, red; C, gray; H atoms) in column (**ii**) along the <001> (**g**), <100> (**h**) and <110> (**i**) zone axes. Red arrows indicate missing linker defects. Scale bars in (**g**–**i**), 2 nm; (**j**) The reconstructed 3D electrostatic potential map viewed in two different orientations, with H atoms omitted for clarity; (**k**,**l**) Missing cluster defects viewed from the <001> direction, adopting the **reo** (**k**) and the **scu** structure (**l**). Columns (**i**) and (**ii**) are CTF-corrected HRTEM images and the projected structural model (Zr, cyan; O, red; C, gray; H atoms). Red dashed boxes show the unit cells. Scale bars, (**k**,**l**), 5 nm. Reprinted with permission from [14]. Copyright 2019, Springer Nature; and (**m**–**o**) Guest species and host MOF: Mn_12_Ac@NU-1000(Zr). (**m**) HRTEM image and ED pattern (inset) of Mn_12_Ac@NU-1000 taken along the <001> zone axis of NU-1000. White arrows point to the Mn_12_Ac clusters. (**n**,**o**) Enlarged images of the highlighted areas in areas 1 and 2 in (**m**), respectively. Reprinted with permission from [77]. Copyright 2019, American Chemical Society.

The octahedral MIL-101(Cr) was almost the first typical MOF whose structures were investigated by TEM [41]. The structural details were further elucidated as techniques advanced. The highly ordered medium-sized cage stack HRTEM images of MIL-101(Cr) collected 4000-fold magnification with a resolution of 2.5 Å, under Cs and contrast transfer function (CTF) correction under low-dose conditions. Each image stack was composed of 120 frames and each frame had an exposure time of 0.05 s. This corresponded to a total exposure time of 6 s, but the total electron dose was only ~8 e^−^ Å^−2^ [42]. Subsequently, the existence of the sublayer surfaces of MIL-101(Cr) was confirmed by the compelling evidence at the atomic scale. The sublayer surfaces terminated by inorganic Cr_3_(μ_3_-O) trimers underwent a transition to stable {111} surfaces regulated by inorganic polynuclear nodes [9]. For ZIF-8(Zn) with highly ordered and oriented macropores, the single crystalline nature of an ideal sample was verified by HRTEM (Figure 3a–c) [75]. HRTEM images captured by a DDEC camera also identified the crystallinity retention and the pore structure of the UiO-66(Zr) nano-cage single crystal doped with WO_4_^2−^. These discoveries helped guide the tuning method of pore size [76].

The surface and interfacial structures of MOFs particles exert impacts on mechanisms of molecular assembly, which in turn affect the morphologies and structures. For TEM imaged ZIF-8(Zn), at an ultra-low dose of ~4.1 e^−^ Å^−2^ and a high frame rate of 40 fps (120 frames in the exposure time of 3 s), under Cs and CTF correction and using a DDEC camera, the spatial resolution of 2.1 Å was sufficient to resolve the single atomic arrangement of Zn and organic linkers in the framework [43]. Terminations of the {110} surfaces were consistent with the armchair model without reconstruction or macro-defects [43]. However, those of the {011} surfaces were caused by doubly coordinated Zn clusters linked to two other clusters [49]. In a thermally treated UiO-66(Zr), ligand-free and ligand-capped surfaces were revealed as coexisting (Figure 3d–f). The major exposed {111} surface was terminated with BDC linkers, and the small truncation surface exposed Zr clusters at the kink positions between {100} and {111} facets [22]. With regard to the ability to image interfaces by HRTEM using DDEC cameras, it was demonstrated that a {110} coherent interface formed between two assembled ZIF-8(Zn) crystals. Its formation was driven by van der Waals’ attractions or by dipole–dipole interactions. An additional layer of extra ligands existed at the interface, suggesting that no chemical reactions occurred in the direct adhesion of the two ligand-terminated surfaces [43]. In short, TEM explores the surface structures of MOFs, from the mere detection of surface steps to the identification of metal clusters, then to the distinction between metal nodes and organic linkers. The coordination of nodes and linkers can be identified by TEM, for example, UiO-66(Zr) [22] and ZIF-8(Zn) [43], which facilitates the further study of the structure–function relationship of MOFs [18].

The coexistence of the ordered “missing linker” and “missing cluster” defects in UiO-66(Zr) was discovered at sub-Å resolution by a combined technique of low-dose TEM and electronic crystallography (Figure 3g–l). The “missing linker” defects (Figure 3g–j) were notably first identified as a topology of **bcu** net due to the defect-terminating formate ligands. High quality HRTEM images were essential to unambiguously resolve all structural components by 3D reconstruction. They revealed an 8-connected network with Zr6O8 clusters, BDC linkers, and terminating formate groups (Figure 3j). This type was prevalent and robust with prolonged crystallization time and crystal ripening. The “missing cluster” defects (Figure 3k,l) were categorized into **reo** and **scu** structures according to the presence or absence of the face-on BDC linkers surrounding but not connected to the missing clusters. This type appeared only in small regions of a few units of cell size and tended to disappear over time but were more catalytically active. The catalytic activity of UiO-66(Zr) was proposed to be improved by understanding defect characteristics and newly developed techniques to control the evolutionary tendencies [14].

In the early days, the characterization of host–guest systems inevitably involved structural decomposition. In spite of this, HRTEM alone or assisted by ED was sufficient to reveal the size, localization, monodispersity, and anchor stability of the guest. For guests with a typical size in the range of 1–5 nm, the study cases include Cu@MOF-5 [78], Ru@MOF-5 [28], Pd@MOF-5 [28], Pd@MOF-177 [79], Pd@MIL-101 [80], Pd@HKUST-1 [81], and Au@ZIF-90 [20]. Electron tomography made the guests’ distribution within the hosts clearer, thus preventing surface bias and other factors from skewing the results [20,28]. As technologies evolve, host and guest can be preserved while structural details are being imaged. For example, the cryo-TEM strategy allowed the characterization of host MOFs encapsulating gas molecular guests [49]. Single-molecule magnets (SMM) are a promising guest species for MOFs. For their potential application in the next generation of computing technology related to molecular spintronics, nanostructured organization and nanoscale protection in a two-dimensional or three-dimensional networks are required to achieve the read and write process. Using a DDEC camera, HRTEM directly imaged the Mn_12_O_12_(O_2_CCH_3_)_16_(OH_2_)_4_ (denoted as Mn_12_Ac, a kind of SMM) molecules with a uniform size of ~2 nm. Mn_12_Ac clusters were shown to be encapsulated and fitted into the hexagonal channels of mesoporous NU-1000(Zr). The adsorption of isolated molecular guests inside hosts was demonstrated for the first time (Figure 3m–o) [77].

It is essential that the DDEC camera quickly locates the zone axis to minimize electron-beam exposure. The traditional manual system requires switching back and forth between imaging and diffraction modes. This time-consuming process results in the passive and undesirable acceptance of hundreds of electrons per Å^2^. A program [22] was developed to achieve a direct one-step zone-axis alignment for crystals with initial orientations close to the zone axis within 5° at a total dose well below 1 e^−^ Å^−2^. This was accomplished by calculating tilt angles from Laue circles identified from off-axis ED patterns. In addition, to reduce the electron-beam induced specimen motion, a common method is to divide the exposure into stacks of sequential short exposures. When using a DDEC camera, the approach requires the precise elimination of drift between frames. To address this issue, an “amplitude filter” was developed to limit the phase analysis to only “reliable” pixels with high amplitudes [22]. This program contributed to the detection of organic linkers with lower atomic numbers on the surface of UiO-66(Zr). Additionally, the thickness effect is important for the phase-contrast image captured by the DDEC camera, especially for the inorganic samples with irradiation sensitivity. It is often necessary to directly simulate the atomic position and the contrast for an accurate interpretation.

Novel DDEC cameras have been a huge boost for HRTEM. They allow nondestructive visualization for MOFs under well-controlled conditions (Table 1). However, for a reasonable image correction and interpretation, HRTEM images captured by DDEC cameras still require a series of defocused images, as is the case with scintillator-based cameras. The low-electron dose basic principle required by MOFs presents a challenge in the determination of Scherzer defocus, a problem that DDEC cameras are not yet able to overcome.

### 3.3. Traditional STEM and iDPC-STEM

In terms of the interpretation of high-resolution images, the coherent electron beam for TEM mode causes contrast reversion with defocus. A series of defocused images and a subsequent analysis based on crystallographic principles is required to determine the true structural information. In comparison, STEM mode with a convergent electron beam has a higher resolution, due to the incoherent phase scattering imaging. Bragg diffraction is only present in low-angle bright-field (BF) and annual dark-field (ADF) imaging modes. The contrast of ADF images is proportional to one-third power of the atomic number. High-angle annual dark-field (HAADF) produces Z-contrast images where the contrast is proportional to the square of the atomic number. Low atomic number elements are difficult to image due to their small scattering angles. Despite this limitation, STEM images are convincing for the direct identification of elements with a relatively obvious difference in contrast between them in the sample. Therefore, the STEM mode has obvious advantages over the traditional TEM mode in its ability to accurately image non-periodic local structures such as defects, surfaces, interfaces, and deformations in crystals [18,74,92,93], and to analyze chemical elements. [28] The spatial resolution of the STEM mode is also enhanced by the use of Cs correctors [72].

**Figure 4:**
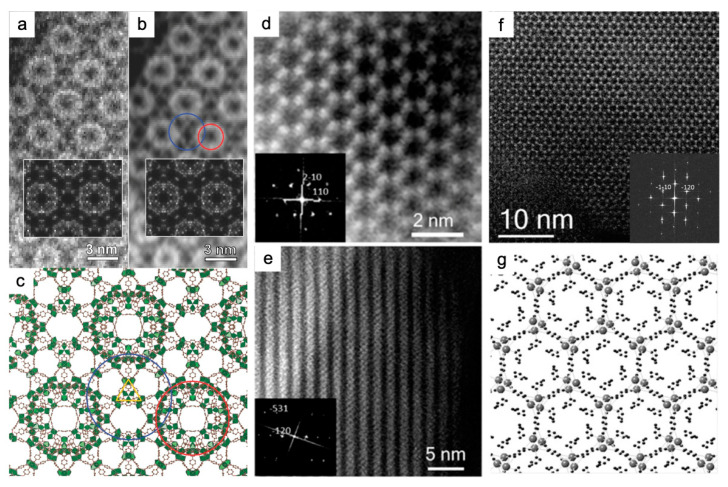
STEM imaging of MIL-101(Cr) [83] and MOF-74(Zn) [40,84]: (**a**–**c**) ADF-STEM images (**a**), low-pass filtered ADF-STEM image (**b**), corresponding simulated images as insets, and structural model (**c**) of MIL-101(Cr) along the <011> direction. Red and blue circles indicate pores with inner pore diameters of 29 Å and 34 Å, respectively. Cr polyhedron, green; C, brown; H and O are not shown for clarity. Reprinted with permission from [83]. Copyright 2016, John Wiley and Sons; (**d**,**e**) Fourier filtered HAADF-STEM images and FFT insets of a MOF-74(Zn) nanoparticle oriented along the <001> zone axis (**d**), and another faced along the <211> zone axis (**e**) with the pores perpendicular to the electron beam. Reprinted with permission from [40]. Copyright 2014, American Chemical Society; and (**f**,**g**) HAADF-STEM images and corresponding FFT of MOF-74(Zn) (**f**) showing the pore structures. (**g**) Structural model (Zn atoms, gray). Reprinted with permission from [84]. Copyright 2015, John Wiley and Sons.

**Figure 5:**
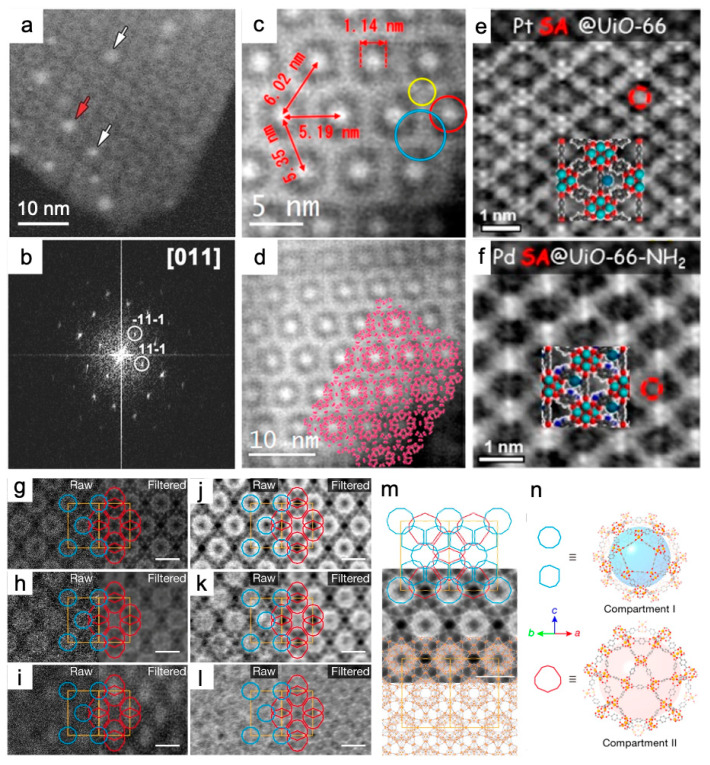
Traditional and iDPC-STEM characterization of host MOFs and encapsulated guest species: Pt@MIL-101(Cr) [83], CsPbI_3_@MIL-101(Cr) [85], Pt@UiO-66(Zr) and Pd@UiO-66(Zr)-NH_2_ [87], TiO_2_-in-MIL-101(Cr) composites [23]: (**a**,**b**) ADF-STEM images and corresponding FFT along <011> zone axis of an MIL-101(Cr) crystal loaded with Pt nanoparticles. The white and red arrows in (**c**) point to nanoparticles at small and large cage positions, respectively. The MIL-101 schematic model are depicted in (**d**). Reprinted with permission from [83]. Copyright 2016, John Wiley and Sons; (**c**,**d**) HAADF-STEM images of MIL-101(Cr) crystal with cavities filled by the perovskite material CsPbI_3_ along the <110> direction. Red, blue, and yellow circles indicate 2.9 nm cage, 3.4 nm cage, and supertetrahedra, respectively. Reprinted with permission from [85]. Copyright 2019, American Chemical Society; (**e**,**f**) iDPC-STEM images of UiO-66(Zr) and UiO-66(Zr)-NH_2_ crystals with encapsulated Pt (**e**) and Pd (**f**) single atoms. Reprinted with permission from [87]. Copyright 2023, American Chemical Society; and (**g**–**n**) HAADF-STEM and iDPC-STEM images of TiO_2_-in-MIL-101(Cr) composites. (**g**–**l**) Raw and filtered HAADF-STEM images (**g**–**i**) and iDPC-STEM (**j**–**l**) images taken from the <110> projection: MIL-101-Cr (**g**,**j**), 23%-TiO_2_-in-MIL-101-Cr (**h**,**k**) and 42%-TiO_2_-in-MIL-101-Cr (**i**,**l**). Scale bars, (**g**–**l**), 5 nm. (**m**) 2D projected potential map (grayscale; **middle**) overlaid with pore arrangement (**top**) and atomic structure (**bottom**). (**n**) Atomic structure and topology of compartment I and compartment II. The positions of the TiO_2_ units corresponding to the mesopores, mesopore I and mesopore II, are outlined in blue and red, respectively. The unit cells are shown in orange. Reprinted with permission from [23]. Copyright 2020, Springer Nature.

The pore structures and super-tetrahedron building blocks of MIL-101(Cr) were observed using ADF-STEM with a beam current of <10 pA and a convergence semi angle of 22 mrad. STEM images validated the controlled synthesis of a well-defined morphology (Figure 4a–c) [83]. The circles in Figure 4c indicate two different pore types: the red one corresponds to a small cage with a diameter of 29 Å, and the blue one to a large cage of 34 Å. With a probe size of 0.8 Å, and a beam current of <1.65 × 10^−10^ A using a convergence semi angle of 17 mrad, the HAADF images provided the “quasi” atomic resolution imaging of nanoscale MOF-74(Zn) prepared at room temperature (Figure 4d,e) [40]. Zn clusters were identified in a hexagonal distribution according to the bright features of the strong scattering factor in the HAADF images. The high crystallinity of MOF-74(Zn) was confirmed by the 6-fold axis shown in the FFT, indexed by a = b = 25.93 Å, c = 6.83 Å, α = β = 90°, and γ = 120°. The probe size was set to 2.5 Å, and the spatial resolution was 0.8 Å (Figure 4f,g) [84]. Cs-corrected HAADF-STEM images visualized layer stackings and identified “missing clusters” defects, which are missing one or a row of clusters, respectively, in 2D Hf-MOFs [48]. Regarding the imaging of host–guest interactions, the ADF-STEM mode with the beam current set to ≤10 pA revealed that the size of Pt nanoparticles is tailored to that of MIL-101 pores. It is demonstrated that the ALD approach was suitable for the uniform deposition of Pt nanoparticles into the pores of MIL-101(Cr), and there was no structural degradation during the loading process (Figure 5a,b) [83]. In addition to metal guests, HAADF-STEM with the beam current down to 2 pA observed the distributions of CsPbI_3_ perovskite QDs, which validated the two-step synthesis at room temperature for encapsulating the QDs in the pores of MIL-101(Cr) (Figure 5c,d). In Figure 5c, the occupying CsPbI_3_ nanoparticles in the pores are seen as 1.14 nm bright circles, which are separated by 5–6 nm. Figure 5d shows the MIL-101(Cr) schematic model superimposed along the <110> orientation. Anomalous contrast revealed that QDs were homogeneously distributed throughout the MOF crystal but did not occupy all the nanocages. This indicated that the MOF crystal can act as a stable reactor for QDs [85]. HAADF-STEM also investigated the anchoring of the W(≡C^t^Bu)(CH_2_^t^Bu)_3_ complex on mesoporous NU-1000(Zr) with high crystallinity using the surface organometallic chemistry (SOMC) method, with a convergence semi angle of 14.9 mrad [94].

Thus, it is a key issue to reduce the structural collapse of hybrid materials such as external defects or lattice damage caused by the high-energy STEM electron beam. The joint use of multiple detectors is preferred to examine comprehensive structural details. In STEM mode, a small electron beam tracks a small target area of the sample, leaving the rest of the crystal is intact. However, especially in Cs-STEM, the highly focused electron beams may burn out the structure of beam-sensitive materials and even form holes. The electron dose and exposure time should be strictly controlled. Traditional methods to reduce the electron dose in STEM mode mainly include decreasing the number of emitted electrons by stepping down the emission voltage of the electron gun and increasing the scanning speed to reduce the dwell time on pixels. Unfortunately, the former is likely to degrade the accuracy of aberration correction, and the latter destabilizes the scanning coil. To further address this issue, the concept of compressive sensing was introduced [74]. An image is represented by a sparse base set with binary random missing pixels and can be recovered at a low sampling frequency. Using line-hopping approximate random adaptive sub-sampling, specimens can be imaged with high resolution and sensitivity, extremely low dose conditions (≤1 e^−^ Å^−2^), and quite fast imaging. Under combined control of the electron dose and the number of electrons per pixel (the beam current), atomic resolution data on crystal structure can be obtained.

**Figure 6:**
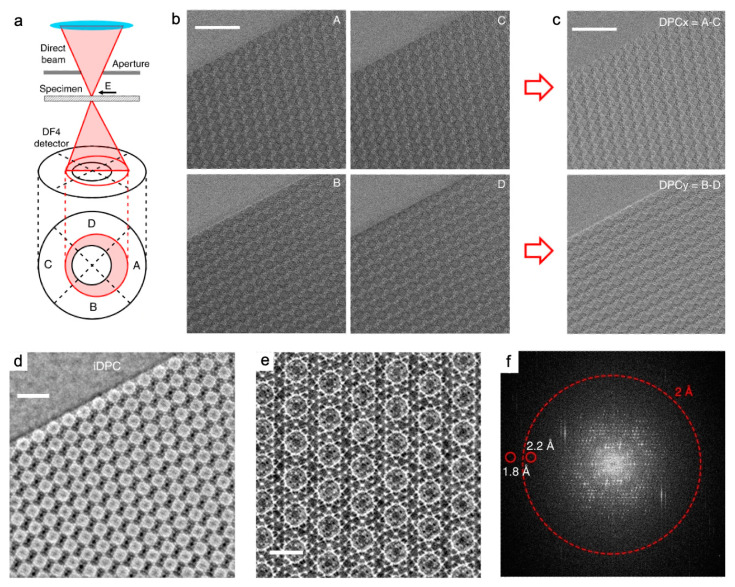
iDPC-STEM schematic set-up and exemplary images of MIL-101(Cr) along <110> projection [18]: (**a**) The schematic set-up of iDPC-STEM. The electron beam is deflected by the potential field (E) in the samples and detected by the four segments (A–D) of the DPC detector; (**b**) Four images each detected by the four segments of the DPC detector, respectively. Scale bar, 20 nm; (**c**) The DPC image obtained from the four images in (**b**). Scale bar, 20 nm; (**d**) The iDPC-STEM image obtained by a 2D integration of the DPC image in (**c**). Scale bar, 10 nm; (**e**) The magnified iDPC-STEM image perfectly matched the structural model. Scale bar, 5 nm; and (**f**) The corresponding FFT pattern of (**e**) in a log scale with an information transfer up to 1.8 Å. Reprinted with permission from [18]. Copyright 2020, Springer Nature.

To achieve a lower dose, higher signal-to-noise ratio, and better contrast, an emerging iDPC-STEM technique offers high efficiency in collecting electron signals. It is equipped with four-quadrant segmented detectors, which can be utilized in the new generation of Cs-STEM without the installation of additional commercial equipment (Figure 6) [18]. This direct electronic phase imaging mode produces the images with the contrast approximately linear with the atomic number, reflecting the electrostatic potential information of the lattice projection. Light and heavy elements can be distinguished simultaneously at sub-Å resolution [18,32]. iDPC-STEM has a sufficient electron utilization and filters out of vector field information such as non-integrable noise during the image integration process. These allow iDPC-STEM to achieve a high resolution and signal-to-noise ratio under the condition of extremely low electron dose.

These advantages are comparable to those of (A) BF-STEM and (HA) ADF-STEM under the same low-dose conditions, with damage-free imaging as a prerequisite. In a practical experimental comparison of HAADF-STEM and iDPC-STEM, cages of MIL-101(Cr) were identified with the same resolution of 4.7 Å, using a beam current of 2 pA and a total dose of 54 e^−^ Å^−2^ [86]. HAADF-STEM can serve as a reference to outline the structures [23] because it is hardly affected by the deflection of the crystal zone axis [95], although it has a limited ability to image light atoms (Figure 5g–n). Compared to traditional STEM imaging, iDPC-STEM offers great potential for low-dose imaging of high-crystallinity, beam-sensitive materials such as MOFs [32,96]. The performance of low-dose iDPC-STEM for local structures is comparable to that of DDEC cameras at cryogenic temperatures [18]. Compared to HRTEM images, iDPC-STEM images are highly interpretable due to the direct correlation between the contrast and the atomic number of the elements.

**Figure 7:**
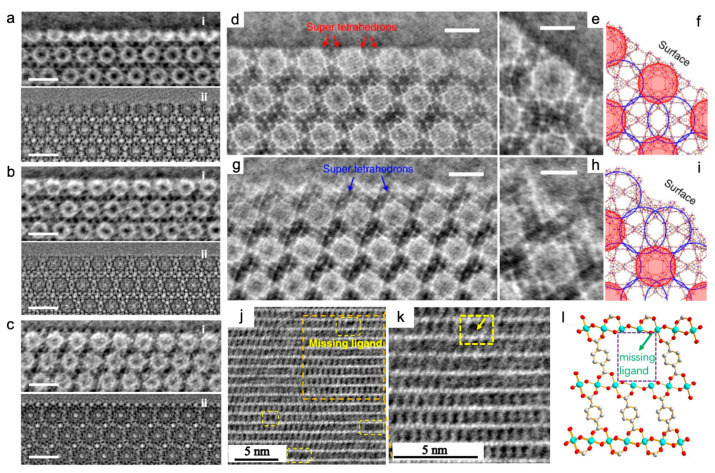
iDPC-STEM imaging of MIL-101(Cr) [18,42] and FeNi-BA-T [97]: (**a**–**c**) iDPC-STEM images (row (**i**) in (**a**–**c**)), and CTF-corrected HRTEM images (row (**ii**) in (**a**–**c**)) of the same sample showing MIL-101(Cr) surface structures. Three vacuum-heated samples using different additives: (**a**) MIL-101-HF (HF), (**b**) MIL-101-Ac (acetic acid), and (**c**) MIL-101-NA (no additive). Scale bars, 5 nm. Reprinted with permission from [42]. Copyright 2019, American Chemical Society; (**d**–**i**) Surface characterizations of MIL-101(Cr). iDPC-STEM images of two types of surface terminations with two types of cages exposed on the {111} surfaces in a MIL-101 crystal: one in (**d**) exhibits the complete characteristic spheres along the {111} surfaces, whereas another in (**g**) is terminated by nearly half of the spheres. Red and blue arrows in (**d**,**g**) indicate super tetrahedrons. The structures of single-unit cells at two types of surface terminations are shown in (**e**) and (**h**), respectively, of which the structural models are shown in (**f**,**i**). Red and blue circles in (**f**,**i**) mark the complete 29 Å and 34 Å cages. Scale bars, (**d**,**g**), 5 nm, (**e**,**h**), 3 nm. Reprinted with permission from [18]. Copyright 2020, Springer Nature; and (**j**–**l**) Missing linker defects in FeNi-BA-T. (**j**) iDPC-STEM images of FeNi-BA-T. (**k**) The enlarged image of the orange dashed box in (**j**). Yellow boxes and the yellow arrow in (**j**,**k**) show the missing ligands. (**l**) The projected structural model of FeNi-BA-T. Reprinted with permission from [97]. Copyright 2022, John Wiley and Sons.

Utilizing the iDPC-STEM technique, the surface cage structure of MIL-101(Cr) was imaged with probe current of 1 pA, an exposure time of 12.6 s, a convergent half-angle of 14.9 mrad, and a collection angle of 16–61 mrad. The total electron dose for each image was as low as ~35 e^−^ Å^−2^. The resolution was slightly lower than the 2.5 Å of HRTEM images, but the contrast was stronger (Figure 7a–c). Three methods, two with respective additives, hydrofluoric acid and acetic acid, and one without additives, were used to prepare the MIL-101(Cr) samples. The uniformity of {111} surfaces and the integrity of the surface cages of the three MIL-101(Cr) showed the significant function of acidic additives in influencing the crystal surface structure of MOFs [42]. In addition, for MIL-101(Cr), the {111} surface of the two crystals was resolved at 1.8 Å by reducing the electron-beam current to less than 0.1 pA (corresponding to an electron flux of 40 e^−^ Å^−2^) and the setting of the convergence half-angle to 10 mrad. The surface-to-surface assembly process of two MIL-101(Cr) crystals was found to be free of organic ligands and lattice mismatches on the attached and connected {111} surfaces. The Cr/BDC super tetrahedrons preferred the 34 Å cage as the energy-stable surface termination, and the adjacent edges maintained the 29 Å cage on the surfaces. A matching interface then formed after the original lattices flipped horizontally and moved about 18 Å (Figure 7d–i) [18]. iDPC-STEM also contributed to the elucidation of the local structure evolution of MIL-101(Cr) under beam irradiation [86].

The “molecular compartment” strategy was newly developed to grow TiO_2_ inside different pores of MIL-101(Cr) and its derivatives. The precise characterization of TiO_2_ locations in real space posed a great influence on understanding of the synergetic mechanisms to enhance the photocatalytic CO_2_ reduction (Figure 5g–n). Pure MOF and TiO_2_-in-MOF composites were characterized with interpretable resolutions of 3.9 Å and 5.2 Å in HAADF images (Figure 5g–i) and increased to 3.2 Å and 3.1 Å in iDPC-STEM images (Figure 5j–l). iDPC-STEM images for light element contrast were more friendly and helped to determine the exact location of mesopores in the MOF and the filling of TiO_2_ units relative to the MOF lattice. Figure 5n highlights compartment I based on mesopore I of 29 Å and compartment II based on mesopore II of 34 Å [23]. Introduction of lattice strain, unsaturated metal sites, and defects were revealed by iDPC-STEM with the beam current <1 pA. This validated the mechanism of increase in OER activity of Ni-BDC by incorporation of Fe^3+^ and 2-aminoterephthalate (ATA) (Figure 7j–l). As shown by the yellow arrow and dashed boxes in Figure 7j,k, “missing linker” defects were observed in the thermal-treated multivariate MOFs. The defects were thought to be caused by the removal of ATA. This MOF was denoted as FeNi-BA-T [97]. It is worth mentioning that the iDPC-STEM technique has enabled the simultaneous imaging of the MOFs and encapsulated single metal atoms [87]. iDPC-STEM visualized the host UiO-66(Zr) and the guest Pt or Pd single atoms in the meantime with a beam current <0.1 pA. Atomic-scale details revealed the adsorption sites of the single atoms: the single Pt atom was located on the benzene ring of the BDC ligand in Pt@UiO-66, while the single Pd atom was absorbed by the BDC ligands. The metals in Pt@UiO-66(Zr) and Pd@UiO-66(Zr)-NH_2_ were presented as clusters. Thus the amino group did not always facilitate the formation of single-atom catalysts (Figure 5e,f) [87].

Spectroscopic analysis based on high-resolution imaging is a powerful tool to probe the chemical composition of samples at the microscopic scale. STEM-EDS (EDS: energy dispersive spectroscopy) and STEM-EELS (EELS: electron energy loss spectroscopy) have been effectively applied to MOFs materials such as UiO-66(Zr) [98]. However, the conventional elemental information acquisition procedure takes a long time, which aggravates the degradation of MOFs. Under cryogenic conditions, damage-free and monochromated STEM-EELS was performed on MIL-100(Al), MIL-100(Fe), and UiO-66(Zr) at an energy resolution of 7 meV, a low electron flux of 10 e^−^ Å^−2^ and a convergence semi angle of 10 mrad, using a DDEC camera. By monitoring the evolutions of characteristic peaks with the controlled electron dose (from 10 e^−^ Å^−2^ to 10^4^ e^−^ Å^−2^), coordination bonds were studied, and chemical group distributions with their intact and degraded parts were discovered in the energy range. These characteristics were then mapped with a spatial resolution of 10 nm. This research provided a methodological reference for the analysis of the chemical properties of beam-sensitive materials in the wide energy range, including infrared (IR), ultraviolet (UV), and X-ray intervals [88].

### 3.4. Dynamic Visualization by In Situ TEM

#### 3.4.1. In Situ Synthesis

The liquid phase is one of the important synthetic conditions for MOFs, where chemical interactions typically generate a large number of tiny intermediates that quickly reach an equilibrium [99]. By directly visualizing their nucleation, growth, and self-assembly dynamics, in situ LCTEM provides insights into MOFs synthesis in solution. It is feasible to investigate the liquid-phase synthesis conditions and in-depth mechanisms through the contrast, morphology, and growth behavior of MOFs. Synthesis conditions such as concentration, temperature, and node to linker ratio can be subsequently verified by ex situ TEM. Given the beam-sensitivity of MOFs, emphasis should be placed on the electron tolerance and methods to reduce damage to the particles to be synthesized in liquid. Furthermore, the surface chemistry of the LC viewing membrane should be controlled together with the electron-beam conditions.

**Figure 8:**
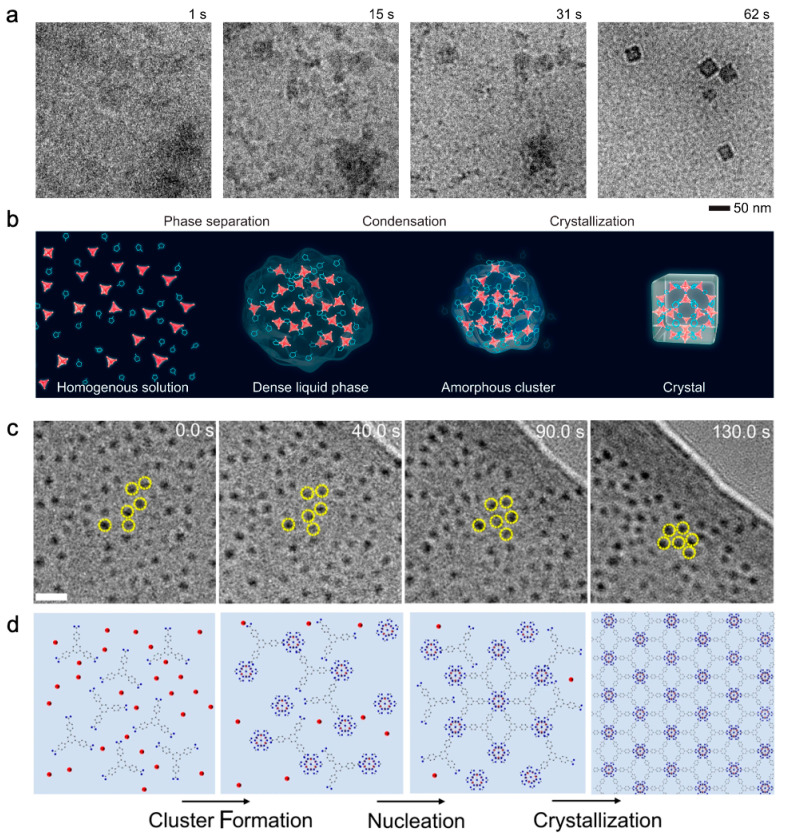
In situ LCTEM imaging unravelling the formation pathways of ZIF-8(Zn) nanocubes [47] and 2D Hf-MOFs [48] from solution: (**a**,**b**) Time series of in situ LCTEM images (**a**) and the schematic illustration (**b**) of the formation process of ZIF-8(Zn). The red and blue shapes represent Zn^2+^ ions and 2-methylimidazole (2-Melm), respectively. Reprinted with permission [47]. Copyright 2021, National Academy of Science; and (**c**,**d**) Time series of in situ LCTEM images (**c**) and the schematic illustration (**d**) of the formation process of 2D Hf-MOFs. The yellow dashed circles in (**c**) highlight the self-assembly process of Hf-clusters. The red, blue, and black atoms in (**d**) represent Hf, O and C, respectively. Scale bar, 10 nm. Reprinted with permission from [48]. Copyright 2022, Springer Nature.

As for macromolecular self-assembled materials, LCTEM was performed at the very beginning to visualize the dynamics of ZIF-8(Zn), which allowed for their low-temperature formation. The cumulative dose is ~20 times less than the damage threshold of ~4000 e^−^ nm^−2^. Nucleation was reproducibly controlled by physical stirring instead of an electron beam when monomers were continuously fed into the LC and was limited by the local consumption of the monomer in the solution. The growth was a surface reaction limited process in which the growth rate and the particle size were controlled by the metal to ligand ratio rather than particle coalescence [44]. Later, the nucleation of ZIF-8(Zn) was recorded and was considered to follow a three-step nonclassical nucleation pathway (Figure 8a,b). By mass of solute concentration, the solution (t = 1 s) phase separated into solute-rich (dark gray contrast) and solute-poor (lighter gray contrast) regions (t = 15 s). The solute-rich regions condensed into an amorphous aggregate (t = 31 s). The aggregate then crystallized into ZIF-8 nanocubes (t = 62 s). Images were captured by the DDEC camera at a rate of 20 frames per second with the electron dose rate controlled to ≤0.05 e^−^ Å^−2^ s^−1^. Images were averaged every five consecutive raw frames to improve signal-to-noise ratio [47].

Metal–organic layers (MOL, referred to as 2D MOFs) have attracted attention due to the merits of 2D materials and MOFs. The multi-step nucleation process of Hf-MOL revealed that Hf-clusters were first formed in solution, then were complexed with ligands to form amorphous precursors, followed by the assembly of cluster-ligand complexes into hexagonally arranged nuclei. The addition of clusters to the surface edges may have served as a pathway for the subsequent growth. To determine this formation pathway, high-quality imaging of organic ligands was important (Figure 8c,d) [48].

It is fascinating to explore the properties and applications of metal–organic nanotubes (MONTS, referred to 1D MOFs), which also exhibit tunable interconnected networks. LCTEM provides opportunities to elucidate the formation mechanisms of discrete or small bundles of tubes. With respect to [**(L1)Cu_2_Br_2_**] (**L1** = 1,4-bis((4H-1,2,4-triazol-4-yl)methyl)naphthalene, a new ligand), initial nucleation events failed to be captured due to limited imaging contrast and resolution at the electron dose of <10 e^−^ Å^−2^. The growth of [**(L1)Cu_2_Br_2_**] at 85 °C was found to be more than three times faster than at 23 °C, using the heating capability provided by the LCTEM chip. The thermodynamically driven process was depicted as a surface-specific monomer–monomer attachment. The precursor ions or continuous amorphous clusters continuously underwent anisotropic growth to form MONT crystals after determining the lowest energy surface for crystal growth [73]. This research group then investigated the growth of Ag-MONTs which were also based on L1 ligands. Unlike the previously studied systems [73], such MONTs crystals formed via multiple pathways depending on the reaction conditions. In a comparatively short amount of time, the precursor ions aggregated and formed short-range clusters to minimize energy. This resulted in primary particles that were non-homogeneously nucleated and developed into anisotropic MONTs bundles as supersaturation progressed [89].

However, it is still difficult to perform ED on MOFs in a liquid environment because of the background signal and electron scattering from the liquid layer, as well as the fluctuation of small specimens. ED can perform postmortem after drying the liquid or by plunge freezing the reaction solutions at the time of interest [47].

#### 3.4.2. Phase Transition

The topological structures of MOFs diversify due to the coordination between organic linkers and metal clusters. For example, Zr_6_-MOFs are composed of [Zr_6_(μ-O)_4_(μ-OH)_4_]^12+^ clusters and multi-host carboxylate-based organic linkers, with 10 topological structures and pore structures [90]. The design of pure-phase MOFs requires an understanding of the conditions and mechanisms that govern the transitions between the multiple phases. The phase transition of Zr_6_-MOFs from microporous **scu**-NU-906 with a characteristic lattice spacing of ~1.7 nm to mesoporous **csq**-NU-1008 of ~3.5 nm was directly observed at 80 °C using LCTEM with a flow cell, with the cumulative electron dose <6 e^−^ Å^−2^ [90]. The process followed the dissolution–reprecipitation mechanism and could be tuned by the formic acid concentration and reaction time. The principle was demonstrated as follows. Formic acid promoted the dissolution of NU-906 and acted as a regulator for the formation of the second phase NU-1008. The dissolution of NU-906 produced an intermediate solution containing Zr_6_ clusters, TCPB-Br_2_ linkers, excess formic acid, and DMF. The composition was the same as the initial synthesis solution of NU-1008, so that the dissolution of NU-906 simultaneously produced NU-1008.

#### 3.4.3. Pore Breathing

The periodic frameworks of MOFs tend to experience reversible lattice transformations, a process known as pore “breathing”, when external factors such as temperature and pressure fluctuate or when guests are adsorbed and desorbed. It is entitled to be gas storage and separation platforms for H_2_O vapor, N_2_, O_2_, CO_2_, CH_4_, H_2_, CO, NH_3_, etc. [100,101]. In situ ETEM directly visualized the lattice changes of MIL-53(Cr) induced by the adsorption and desorption of H_2_O molecules on it during temperature cycles from heating to 300 °C to cooling to 27 °C, with a cumulative dose of ~5 e^−^ Å^−2^. This pore breathing was originally triggered by the adsorption of the first single H_2_O molecule per unit cell of MIL-53(Cr) at 300 °C. These initially adsorbed H_2_O molecules were anchored by hydrogen bonds formed between them and the bridging μ-OH groups of MIL-53(Cr) during this activation process. They remained stable throughout the following temperature-modulated adsorption and desorption [91].

#### 3.4.4. On Demand Structural Modification

Damage-free characterization requires the determination of the threshold of the cumulative electron dose threshold at which MOFs begin to collapse. However, structural modification may occur before it is detectable. One way to maximize the integrity of MOFs is to quantitatively characterize the local structural evolution under in situ irradiation and its underlying mechanisms.

**Figure 9:**
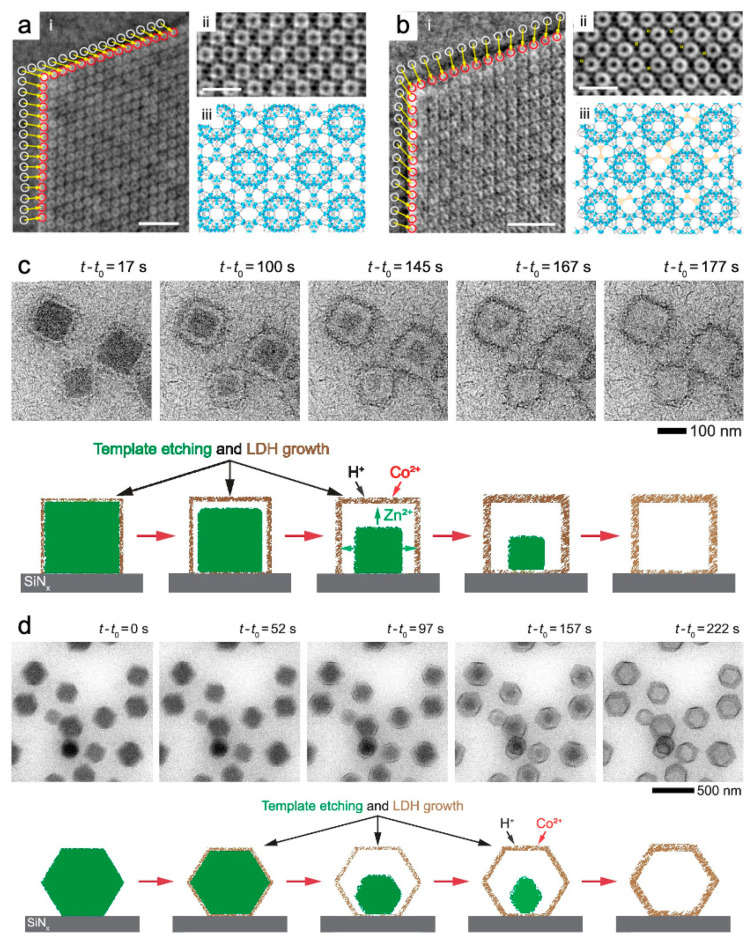
In situ structural modification of MIL-101(Cr) [86] and ZIF-8(Zn) [102]: (**a**,**b**) In situ irradiation of MIL-101(Cr) crystals. (**i**) in (**a**,**b**) are iDPC-STEM images before and after beam irradiation, respectively. White and red circles mark the initial and final positions of the pores at the crystal edge. Scale bar, 50 nm. (**ii**) in (**a**,**b**) are the zoomed-in views of iDPC-STEM images. In (**b**), yellow boxes in (**ii**) indicate the possible displacement of super tetrahedrons. Scale bar, 10 nm. (**iii**) in (**a**,**b**) are the illustration of the displacement of super tetrahedrons. Reprinted with permission from [86]. Copyright 2020, American Chemical Society; and (**c**,**d**) In situ conversion of ZIF-8 nanocubes (**c**) and rhombic nanododecahedrons (**d**) into LDH nanocages in liquid phase. Time series of in situ LCTEM images and corresponding schematic illustrations are provided respectively. *T*_0_ represents the time point when the etching was first visually detected. Reprinted with permission from [102]. Copyright 2021, American Chemical Society.

The local evolution of the pores of MIL-101(Cr) single crystals was observed at a resolution of 4.7 Å using the iDPC-STEM technique. It was found to depend on both the crystal plane and the specific position in the crystal (Figure 9a,b). At the molecular level, importance was attached to super tetrahedrons, which were composed of μ_3_O-bridged trimetric chromium oxide clusters and organic linkers. Through quantitative irradiation analysis, crystal shrinkage and deformation with increasing in electron dose was attributed to the molecular displacement and asymmetric component distribution. These were inhomogeneous within the single MOF crystal [86]. The structural evolution of ZIF-L(Zn) was captured using a DDEC camera at a dose rate of 0.25 e^−^ Å^−2^ s^−1^ (20 frames with 0.5 s/frame exposure). Notably, according to the accumulated electron flux threshold of 100 e^−^ Å^−2^ s^−1^, the electron beam-induced degradation of ZIF-L(Zn) was divided into two stages. The first stage was the widely reported structural damage, manifesting as significant deformation and amorphization. The second stage was the molecular breakdown of the organic linker 2-methyl-imidazole (2-mIm). It severely affected the dielectric function and the energy of electronic transitions as determined by core-loss EELS C and N K-edge analysis [82].

Long-range ordered MOFs crystals are suitable as templates for the formation of monodisperse particles and clusters and other nanomaterials, due to their uniform internal pore structures. To this end, TEM was used to rapidly degrade MOFs to form Ag nanoclusters of few to tens of atoms in size [103] and Cu nanoparticles of tunable size from a few nanometers to a few hundred nanometers [104]. The in situ heating sample holder enabled and visualized the formation of Ni nanoparticles from carbonization of Ni-MOFs. The transformation process began significantly at 400 °C, and Ni nanoparticles tended to aggregate and entrap in the carbon matrix after reaching 700 °C. This was due to the increased crystallinity of the Ni nanoparticles and the stability of the carbon matrix at high temperatures [105]. The structural transformation of ZIF-67(Co) during pyrolysis was monitored, the derivatives of which served as ideal ORR catalyst templates. ZIF-67(Co) crystals underwent an ordered–disordered transition at 442 °C, with precipitation of Co atomic clusters and a loss of nitrogen. This was possibly due to the pyrolysis of CoN_4_ tetrahedra above 500 °C, followed by considerable carbonization above 800 °C [106]. Other research groups discovered that Co nanocrystals were uniformly dispersed in the early stages of carbonization. However, as the temperature increased, they became larger and moved toward the carbon surface. Some of the tiny Co nanocrystals which escaped after heating to 1000 °C volatilized, while others catalyzed the formation of carbon nanotubes. During pyrolysis, the carbon texture started to crystallize at 600 °C and was completely transformed at 800 °C [107]. Hollow layered double hydroxide (LDH) materials are promising in the field of catalysis and energy storage. ZnCo(OH)*x*, a kind of LDH, was shown through LCTEM to be formed based on continuous etching and similar growth rate of ZIF-8(Zn) and ZIF-67(Co)@ZIF-8(Zn) (Figure 9c,d). Nanocubic and nanorhombic dodecahedrons were selected to effectively capture the dynamics. Their well-defined shapes made it easy to identify the reaction front, and the electron dose rate was set to <0.1 e^−^ Å^−2^ s^−1^ [102].

## 4. Conclusions and Outlook

MOFs, promising and unique in various applications, are susceptible to electron-beam irradiation by means of powerful but challenging TEM methods. Key experimental considerations for the structural integrity of MOFs under electron irradiation include low electron dose and temperature. Traditional TEM-based methods limit the research possibilities of MOFs. Fortunately, groundbreaking discoveries have been made thanks to the advancement of new technologies and instrumentation based on TEM, including 3DED, DDEC camera, and iDPC-STEM. Static structural characterization unravels details of surfaces, interfaces, defects, and host–guest interactions. Dynamic exploration provides insights into the mechanisms of MOFs formation, phase transitions, pore breathing, and on-demand structural modification under electron-beam irradiation.

Research advances in imaging MOFs using TEM are listed in Table 1, including key techniques, experimental conditions, and characterized structures. Regarding two fundamental concepts: (1) low-dose TEM: This is a guideline to be followed. It inspired the advanced DDEC cameras used in TEM imaging mode and the newly-developed iDPC-STEM technique. The goal is to direct image beam-sensitive MOFs with high resolution and a high signal-to-noise ratio at an ultra-low electron-beam dose and (2) cryo-TEM: Regarding the application scope of the three typical techniques. (1) ED: It is mainly used to characterize periodic structures. Investigations of non-periodic structures are less common. Nonetheless, local structures have been characterized by the 4D-STEM-based SED technique. Missing cluster defects have been identified and guest species in the frameworks have been localized using 3DED technique. It is worth mentioning that in addition to direct imaging, an in situ TEM method was performed in combination with ETEM and 3DED; (2) Imaging in TEM mode: The electron utilization during imaging is effectively maximized by DDEC cameras. The structural interpretation is facilitated by CTF corrections; and (3) Imaging in STEM mode: Image contrast can be used directly to identify the relative atomic numbers of elements. The advanced iDPC-STEM technique can simultaneously image both light and heavy elements while improving electron utilization. Traditional STEM imaging techniques, including (HA)ADF, are complementary to structural analysis.

Further in-depth research on MOFs is expected to benefit from the continued development of current techniques, such as the performance of cameras to acquire and utilize electron signals, and the imaging capability of low-voltage TEM. Furthermore, it is anticipated that ever-evolving and innovative TEM-based techniques will be adaptable to MOFs. They may even reveal previously unobservable details. For instance, electron tomography [108] may help reconstruct the atomic-scale structure of MOFs alone or in combination with their guest species without sacrificing MOFs frameworks. Additionally, the data obtained from the reconstruction of the large-scale data acquired by 4D-STEM [109] provides information on stress and strain, as well as electric and magnetic field distributions, in addition to structures. Additionally, TEM imaging often requires samples with thicknesses <100 nm in order to reduce the multiple scattering of electrons in the sample to satisfy the weak-phase-object approximation (WPOA), although typically only the pseudo weak-phase-object approximation (PWPOA) is fulfilled. Sample thickness is also a challenge for low-dose imaging with high spatial resolution. However, the intrinsic size of the MOFs that need to be studied may not meet this criterion, but this can be improved by using sample-protective preparation methods and state-of-the-art imaging techniques. For the former, it is expected that cryo-focused ion beam (cryo-FIB) [110,111] will be used to prepare MOFs crystals with sizes beyond the nanoscale into samples which are adaptable to TEM observation. This is due to the micro- and nano-processing capability of the ion beam and the protection of MOFs samples from ion-beam damage when processed under cryogenic conditions. However, such research is still in its infancy due to the difficulty of the currently practical methods in meeting the necessary expectations, but it shows great potential for technological advancement. Among the latter, the multislice electron ptychography method is promising for MOFs. This technique is based on 4D-STEM, which combines STEM and 2D coherent ED patterns. It has been successfully used to measure the atomic structure of zeolite, a type of material that is also sensitive to electron beams [112,113]. ZSM-5 up to 40 nm thick was imaged with a lateral resolution of ~0.85 Å and a depth resolution of ~6.6 nm. Individual O atom columns and phase boundaries were resolved. Resolving power of this method within the beam-sensitive specimen and along the projection direction exceeded that of iDPC-STEM technique [113]. An incorporated adaptive propagator addressed the impact of specimen misorientations on the accuracy and reliability of the image [95].

It is envisioned that the novel and emerging TEM techniques will continue to provide critical understanding as essential characterization approaches and research platforms for MOFs.

## Figures and Tables

**Table 1:** Examples of imaging MOFs using advanced TEM-based techniques. [**(L1)Cu_2_Br_2_**] is a kind of 1D MOFs based on a new ligand **L1**.

Imaging Techniques	Advanced Cameras	Materials	Accelerating Voltage	Temperature	Damage Threshold of Electron Dose	Cumulative Electron Dose for Imaging	Spatial Resolution	Imaged Structures	Reference	Year
HRTEM		MOF-5(Zn)	80 kV	Cryo (liquid nitrogen temperature)				Surface	[29]	2012
HRTEM		2D Cu_2_(TCPP) film	80 kV	Cryo				Bulk	[45]	2021
HRTEM	DDEC camera	ZIF-8(Zn)	300 kV		~25 e^−^ Å^−2^	4.1 e^−^ Å^−2^	2.1 Å	Bulk, surface, interface	[43]	2017
HRTEM	DDEC camera	ZIF-8	300 kV			~5 e^−^ Å^−2^		Bulk	[22]	2018
HRTEM	DDEC camera	ZIF-8(Zn)	300 kV					Bulk	[75]	2018
HRTEM	DDEC camera	ZIF-8(Zn), CO_2_@ZIF-8(Zn)	300 kV	Cryo (~103 K)	~50 e^−^ Å^−2^	~7 e^−^ Å^−2^		Bulk, surface, host–guest interactions	[49]	2019
HRTEM	DDEC camera	protein-ZIF-8(Zn)	200 kV	Cryo		1 e^−^ Å^−2^ s^−1^ dose rate and 5 s exposure time)		Nucleation, growth	[50]	2020
HRTEM	DDEC camera	MIL-101(Cr)	300 kV		~16 e^−^ Å^−2^	~8 e^−^ Å^−2^	2.5 Å	Bulk, surface	[42]	2019
HRTEM	DDEC camera	MIL-101(Cr)	200 kV		22–32 e^−^ Å^−2^	10 e^−^ Å^−2^		Bulk, sublayer surface, surface	[9]	2020
HRTEM	DDEC camera	UiO-66(Zr)	300 kV		10–20 e^−^ Å^−2^	~12 e^−^ Å^−2^		Bulk, surface	[22]	2018
HRTEM	DDEC camera	UiO-66(Zr)	300 kV					Surface, defects	[14]	2019
HRTEM	DDEC camera	HKUST-1	300 kV			~6 e^−^ Å^−2^		Bulk	[22]	2018
HRTEM	DDEC camera	W doped UiO-66(Zr)	200 kV			5–10 e^−^ Å^−2^		Bulk	[76]	2018
HRTEM	DDEC camera	Mn_12_Ac@NU-1000(Zr)	300 kV					Bulk, host–guest interactions	[77]	2019
HRTEM	DDEC camera	ZIF-L(Zn)	300 kV		~26 e^−^ Å^−2^			Structural modification	[82]	2021
ADF-STEM		MIL-101(Cr), Pt@MIL-101(Cr)	200 kV					Bulk, surface, host–guest interactions	[83]	2016
HAADF-STEM		MIL-101(Cr), TiO_2_-in-MIL-101(Cr) composites	300 kV				3.9 Å, 5.2 Å	Bulk, surface, host–guest interactions	[23]	2020
HAADF-STEM		MOF-74(Zn)	300 kV					Bulk	[40]	2014
HAADF-STEM		MOF-74(Zn)	300 kV				2.9 Å	Bulk, surface	[84]	2015
HAADF-STEM		CsPbI_3_@ MIL-101(Cr)	300 kV					Bulk, surface, host–guest interactions	[85]	2019
HAADF-STEM		MIL-101(Cr)	300 kV			54 e^−^ Å^−2^	4.7 Å	Bulk, surface	[86]	2020
HAADF-STEM		ZIF-L(Zn)	200 kV		~25 e^−^ Å^−2^			Structural modification	[82]	2021
HAADF-STEM		2D Hf-MOFs	300 kV		106 e^−^ Å^−2^			Bulk, surface, interface, defects	[48]	2022
iDPC-STEM		MIL-101(Cr)	300 kV			~35 e^−^ Å^−2^		Surface	[42]	2019
iDPC-STEM		MIL-101(Cr)	300 kV			<40 e^−^ Å^−2^	1.8 Å	Bulk, surface, interface, defects, nodes and linkers	[18]	2020
iDPC-STEM		MIL-101(Cr), TiO_2_-in-MIL-101(Cr) composites	300 kV				3.2 Å, 3.1 Å	Bulk, surface, host–guest interactions	[23]	2020
iDPC-STEM		MIL-101(Cr)	300 kV			54 e^−^ Å^−2^	4.7 Å	Bulk, surface	[86]	2020
iDPC-STEM		protein-ZIF-8(Zn)	300 kV	Cryo		30 e^−^ Å^−2^		Bulk, nucleation, growth	[51]	2022
iDPC-STEM		Pt@UiO-66(Zr), Pd@UiO-66(Zr)	300 kV				~4.7 Å	Bulk, host–guest interactions	[87]	2023
STEM-EELS	DDEC camera	MIL-100(Al), MIL-100(Fe), UiO-66(Zr)	100 kV	Cryo (125 K)		10 e^−^ Å^−2^	10 nm (energy resolution: 7 meV)	Chemical information	[88]	2023
In situ TEM: liquid cell		ZIF-8(Zn)	200 kV, 300 kV		~4000 e^−^ nm^−2^			Nucleation, growth	[44]	2015
In situ TEM: liquid cell	DDEC camera	ZIF-8(Zn)	200 kV		5 e^−^ Å^−2^			Nucleation, growth	[47]	2021
In situ TEM: liquid cell, heating	Advanced scintillator-based camera	1D [(L1)Cu_2_Br_2_] MOFs	300 kV	Room temperature (23 °C), heating (85 °C)		<10 e^−^ Å^−2^		Nucleation, growth	[73]	2019
In situ TEM: liquid cell	DDEC camera, advanced scintillator-based camera	1D Ag-MOFs	300 kV		70 e^−^ Å^−2^			Nucleation, growth	[89]	2020
In situ TEM: liquid cell, heating	DDEC camera, advanced scintillator-based camera	NU-906, NU-1008	300 kV	Room temperature, heating (80 °C)		<6 e^−^ Å^−2^		Bulk, phase transition	[90]	2020
In situ TEM: ETEM (gas)	Advanced scintillator-based camera	H_2_O@MIL-53(Cr)	300 kV	Room temperature (27 °C), heating (800 °C)		~5 e^−^ Å^−2^		Pore breathing	[91]	2017

## Data Availability

Not applicable.
